# Climate change and youth development: A view of an emerging field

**DOI:** 10.1177/01650254251317141

**Published:** 2025-02-11

**Authors:** Sander Thomaes

**Affiliations:** Utrecht University, The Netherlands

**Keywords:** Climate change, youth/adolescence, behavior change, climate anxiety, environmental education, cross-cultural research

## Abstract

Climate change is a defining challenge of our time, and it disproportionally impacts young people. This poses a call to action for developmental science. How does climate change shape youth’s psychological development and well-being? Can we use our expertise to empower youth to cope with and help mitigate climate change? The emerging field of research on climate change and youth development addresses these timely questions. Here I provide a concise perspective on the field, highlighting lines of research and ideas, including our own, that have begun to develop in recent years. Climate change threatens our global society, which means that our research should be global as well. I call for coordinated, international, and cross-cultural investigation to address the big questions ahead of us and empower young people from across the globe to respond to the challenges of a warming world.

Today’s young people are the first generation to come of age in a world threatened by climate change (Sabarwal et al., 2024; Sanson et al., 2019; United Nations International Children’s Emergency Fund, 2021). This poses a call to action for developmental science. What are the psychological and developmental consequences of climate change for young people? Can we use our expertise to provide deeper understanding of young people’s pro-environmental (i.e., sustainable, climate-relevant, or “green”) attitudes and behaviors? Can we find science-based solutions to empower young people in their efforts to cope with and help mitigate climate change? The emerging field of research on climate change and youth development addresses these timely questions. Here, I will provide a perspective on the field, focusing on research conducted with children and adolescents (roughly below the age of 25). The goal is not to provide a comprehensive synthesis of all research in the field. Rather, the goal is to provide an accessible review of research findings and ideas, including our own, which I hope will encourage future efforts to understand and support youth development amid climate change.

## Youth Climate Activism

Young people cannot be held accountable for providing solutions to climate change. Still, they have become a vital force at the center of the climate movement. Across the world, environmentally engaged youth have led climate protests, successfully capturing the attention of the public and the powers that be (De Moor et al., 2020; Han & Ahn, 2020). They have also filed lawsuits demanding governments and industries to take action on climate change (Kotzé & Knappe, 2023; Parker et al., 2022), participated in climate policy networks and political processes (Kolleck & Schuster, 2022; O’Brien et al., 2018), launched climate campaigns on social media (Belotti et al., 2022; Boulianne et al., 2020), or engaged in community-based climate actions (Bandura & Cherry, 2020; Trott, 2019). While there are multiple ways in which young people can engage in climate activism (e.g., depending on their abilities, resources, and contexts they grow up in), the function of climate activism generally is the same: to voice concern about climate change, express dissent against the status quo, and act for change (Neas et al., 2022; O’Brien et al., 2018).

Youth-led activism is not just inspiring or hopeful, it is also fascinating from a developmental perspective. Even if only a minority of youth engage in climate activism, this minority defies the popular stereotype suggesting that young people, especially adolescents, are apethetic, narrowly self-interested, or subject to the tyranny of the now (Cammaerts et al., 2014; Romer et al., 2017). Youth activists do not just stand up for the climate out of self-interest—they also do so out of interest with people whom they have never met, who live in far away parts of the world, or who are yet to be born. Philosopher Roman Krznaric argues that many of today’s societal problems could be solved if we would regain our imaginative skill to care and plan for future generations (Krznaric, 2020). Young people standing up for the climate demonstrate such a skill: In Krznaric’s words, they are acting as “good ancestors.”

What are the psychological and developmental forces that motivate youth climate activism? For one, engaging in activism can serve as an antidote to experiencing anxiety and despair, providing youth with a sense of determination, purpose, and solidarity instead (Sanson & Bellemo, 2021). Qualitative studies, based on in-depth interviews with young climate activists, provide additional insight into what motivates youth climate activism (De Moor et al., 2020; Fisher, 2016; Haugestad et al., 2021; Van de Wetering & Lee, 2025). The collective picture that emerges from these interviews is that engaging in climate activism aligns well with adolescents’ core developmental needs and motives. One example is adolescents’ need or motive to find out “who they are,” pursue self-coherence and self-consistency, and seek distinctiveness from others—that is, to develop identity (Branje et al., 2021; Erikson, 1968; Thomaes et al., 2017). In explaining their motivations for engaging in climate activism, many interviewed youth mention that their activism allows them to express who they are or what they deeply care about. By engaging in activism, they live up to their personal values and ideals. Similarly, they often refer to a sense of shared identity—that is, the realization that together with other activists, they voice the concerns of their generation (even if activism can also lead to a sense of alienation from non-activist others; Conner et al., 2023).

Climate activism also aligns with adolescents’ increased concern for fairness and social justice (Crone, 2013; Daniel et al., 2016; Krettenauer & Victor, 2017). For example, interviewed youth mention the many injustices (e.g., generational or geographical injustices) associated with climate change, and how they are motivated to stand up for what is right. As one adolescent activist, a Dutch girl, said (Van de Wetering & Lee, 2025):

The Global North of course emits more than the Global South, but we are [. . .] not the ones who are most affected in the first place. It can’t be that youth my age don’t get the same opportunities just because they were born [in the Global South].

Finally, climate activism aligns with adolescents’ increased need for autonomy (Soenens et al., 2017; Zimmer-Gembeck & Collins, 2006). Interviewed youth typically talk about their actions as personal and deliberate decisions, resulting from deeply held convictions. As such, youth climate activists can be seen as rebels with a cause—that us, both a collective cause (i.e., a healthier planet for one’s own and future generations) and an individual cause (i.e., the pursuit and fulfillment of core adolescent needs and motives).

## Impacts of Climate Change on Youth Development

Climate change is consequential for young people. Today’s youth need to find their way in a world exposed to climate impacts, which will become worse in the upcoming decades. For example, under current climate policy agreements, today’s youth are projected to experience at least four times more heatwaves during their lifetimes compared with those born in 1960 (Thiery et al., 2021). Similar trends are predicted for the frequency, intensity, and spread of other extreme weather events—together, out changing climate will disproportionally affect young people’s physical and psychological health and opportunities to learn (Sabarwal et al., 2024; Thiery et al., 2021; Watts et al., 2019).

United Nations International Children’s Emergency Fund (2021) has developed the Children’s Climate Risk Index, which indicates the extent to which young people living in various parts of the world (i.e., based on data from 163 countries) are at risk of climate and environmental hazards, including extreme heatwaves, storms, floods, water-scarcity, or vector-borne diseases. One finding from this work was that virtually all children in the world are or will be exposed to at least one of these hazards. As United Nations International Children’s Emergency Fund (2021, p. 111) puts it: “The climate crisis affects or will affect all children, everywhere, in often significant, life-changing ways, throughout their lives.” A second finding was that approximately one billion children—that is, almost half of the world’s children—live in countries that are at an “extremely high risk” from climate and environmental hazards. Children growing up in these countries, mostly in central Africa and South-East Asia, are exposed to multiple hazards at the same time, while being particularly vulnerable due to unreliable access to essential services (e.g., education, health care, or water sanitation) in times of crisis.

Unsurprisingly, climate change impacts the emotional lives and psychological well-being of our youth. In one influential study, a large-scale investigation of climate anxiety, a total of 10,000 young people (ages 16–25) from 10 countries and 6 continents were surveyed (Hickman et al., 2021). In this study, 84% of the respondents indicated to be at least “moderately” worried, if not “very” or “extremely” worried, about climate change. More than half of the respondents indicated that climate change makes them feel anxious, sad, angry, or powerless. Moreover, 45% of the respondents indicated that their feelings about climate change negatively affect their daily life and functioning.

A comprehensive review of the mental health impacts of climate change is beyond the scope of the present article (for such reviews, see Burke et al., 2018; Vergunst & Berry, 2022). What is clear, though, is that climate change can have direct, vicarious, and indirect effects on young people’s mental health (e.g., disturbed sleep, post-traumatic stress, anxiety and mood disorders; Burke et al., 2018; Crandon et al., 2022; Vergunst & Berry, 2022). Direct effects may occur when exposure to extreme weather-related events (e.g., storms, floods) worsen existing mental health problems or increase their risk of onset. Vicarious effects may occur when the existential threat of climate change leads to enduring stresses or worries, eroding mental health. Indirect effects may occur when climate change causes social and economic disruptions (e.g., famine, forced displacement, war) that, in turn, exacerbate existing mental health problems or trigger their onset (Burke et al., 2018; Intergovernmental Panel on Climate Change, 2022; Vergunst & Berry, 2022).

Even when climate change impacts youth mental health, this does not mean that it is inherently maladaptive for young people to experience negative thoughts or feelings about the climate. Rather, these experiences can be seen as understandable and rational responses to a major threat. Moreover, they can motivate youth to take action. Experts have been concerned that the opposite may be true, and that climate anxiety, in particular, can become so intense that it instills a sense of “eco-paralysis” (e.g., The Lancet Child & Adolescent Health, 2021)—that is, a passive state of behavioral stasis, caused by feelings of helplessness or fatalism (Albrecht, 2011; Verplanken et al., 2020). In a recent series of studies, we tested this idea in community samples of Dutch and Colombian adolescents, but found little evidence for it (Becht et al., 2024). Instead, we found that youth who reported relatively high levels of climate anxiety were more (rather than less) likely to engage in a diverse set of pro-environmental behaviors (i.e., behaviors that benefit the environment or harm it as little as possible; Steg & Vlek, 2009). While this effect was rather modest for so-called “private-sphere” pro-environmental behaviors (e.g., saving energy, recycling, sustainable consumption), it was strong for “public-sphere” pro-environmental behaviors (e.g., persuading others to adopt sustainable lifestyles, climate activism). For most youth, climate anxiety is a reasonable and constructive response to a warming world (see Ogunbode et al., 2022, for a similar argument based on international data).

## What Keeps (Some) Youth From Engaging in Pro-Environmental Behavior?

Given that young people are concerned about and disproportionally impacted by climate change, one might expect that, as a group, they are also prone to engage in high levels of pro-environmental behavior. Yet, research suggests otherwise. In fact, research has found evidence suggesting that young people, especially adolescents, show lower rates of private-sphere pro-environmental behaviors as compared to adults (Grønhøj & Thøgersen, 2009; Otto et al., 2019; Wray-Lake et al., 2017). For example, in one study that tested generational differences in pro-environmental engagement, a representative sample of Danish adolescents were on average about 30% less likely to engage in electricity saving or waste recycling behaviors than their parents (a smaller difference was found for consumption of organic products; Grønhøj & Thøgersen, 2009). Thus, while most adolescents are concerned about the climate, evidence suggests that, as a group, their engagement in private-sphere pro-environmental behavior may lag behind. While such concern-behavior gaps are only human, and certainly not unique to adolescents (Gifford, 2011; Lou & Li, 2023), what might explain them?

One explanation is that adolescents can feel unable to meaningfully contribute to climate change solutions—that is, some adolescents experience low environmental self-efficacy or agency. For example, they may be less likely to act on their climate concerns if they realize that human behavior change by itself will not suffice for climate change mitigation, or feel that their parents make all the impactful decisions for them (Baldwin et al., 2023; Sarrasin et al., 2022). Another explanation is that subsets of adolescents experience at least some degree of climate skepticism—that is, they are unsure of the devastating consequences, human causes, or even the existence of climate change (i.e., climate “impact,” ‘attribution,’ and “trend” skepticism, respectively; Lee et al., 2020; Ojala, 2015).

Impact skepticism now appears to be the most common form of climate skepticism among youth. In an ongoing survey, we sampled youth ages 12–14 from the Netherlands, China, and Colombia. The samples (aggregate *N* = 5,244) were nationally representative in terms of gender and age distribution, household size, and region. We asked participants to indicate their agreement with the item “Climate change is as big a problem as researchers claim.” In the Netherlands, 17% of youth doubted that the statement is true, and another 19% indicated to be undecided. Thus, more than one out of three Dutch young adolescents (i.e., 36%) did not unequivocally agree with the statement. In China, this proportion was smaller. A total of 18% of Chinese young adolescents did not unequivocally agree with the statement. In Colombia, the proportion was somewhat smaller still. A total of 11% of Colombian young adolescents did not unequivocally agree with the statement. Note that in the Netherlands, youth mostly learn about climate change vicariously (e.g., through what they learn in the media or at school): the direct impacts they experience from climate change are still mild. In China, many youth do experience climate impacts more directly, such as through air pollution in metropolitan regions that is worsened by climate change (Hong et al., 2019). Similarly, in Colombia, many youth experience climate impacts directly—for example, the recurrence of extreme weather events in Colombia is among the highest in South America, and Colombia has been hit in recent years by devastating floods and landslides (World Bank, 2024). While evidence from more countries is needed, these findings are consistent with the possibility that adolescents’ climate impact skepticism is shaped by how climate change manifests in the country in which they grow up.

In one study (Grapsas et al., 2023), we analyzed data from the same samples, and we found that individual differences in adolescents’ climate skepticism (aggregated across impact, attibution, and trend skepticism) are tied to emerging basic values, as distinguished in Schwartz’s theory of basic values (Schwartz, 2012). Specifically, we found that self-enhancement values, which reflect a person’s orientation toward personal advancement (e.g., comprising power and achievement), predicts higher levels of climate skepticism. Furthermore, we found that self-transcendant values, which reflect a person’s orientation toward communal welfare (e.g., comprising benevolence and universalism), predict lower levels of climate skepticism. These findings are not surprising, perhaps, if one considers that taking climate change and its impacts seriously requires that one prioritizes collective welfare over self-interest. Importantly, we found that this pattern of associations was largely similar across countries, suggesting that adolescents’ value systems may be foundational to how they relate to the threats of climate change.

## Climate Communication and Education

As discussed, the lives of today’s youth will be impacted by climate change, and the reality of climate change can be hard for youth to cope with, fully acknowledge, and respond to. As a field, we need to find means to prepare youth for the uncertain future that they face. Effective climate communication and education will be key. This is especially true in light of the increased availability of fake news and misinformation online, to which youth may be especially vulnerable (Kelly et al., 2022; Lee et al., 2020). How can we communicate with young people about climate change and its solutions, such as in our publicity campaigns, in our schools, or in our families?

In communicating with young people about climate change, it seems to make sense to emphasize the catastrophes that will occur in the future if we fail to act now—indeed, it is critical that we provide youth with honest and accurate information (Davidson & Kemp, 2024; Kelsey, 2016; Obach, 2023). And yet, the question is whether pointing out the risk of climate catastrophes, potentially eliciting a sense of despair or powerlessness, helps youth cope and engage with climate change. Research in this area is still limited. But the available evidence suggests that conveying perspective and hope, rather than doom and gloom, may be more empowering in helping youth respond to climate change (Jones & Davison, 2021; Ojala, 2012; Stafford et al., 2023; Stevenson & Peterson, 2015). Importantly, hope is not just a feel-good emotion—rather, hope can be a power source for coping and action (Marlon et al., 2019; Ojala, 2012). Moreover, hope in the face of climate change is not baseless or utopian. Climate scientists point out that, while our planet is in bad health, there is still reason for optimism (Mann, 2023). Most importantly, we know exactly what needs to be done to halt climate change. What is needed is political will, economic reform, and collective action to create the large-scale change that the world needs now.

One way to create support for such needed change is through widespread climate and environmental education. We conducted a meta-analysis on the effects of such education for children and adolescents (Van de Wetering et al., 2022). Environmental education is an umbrella term for diverse educational activities and programs that aim to increase students’ knowledge of or ability to protect the climate, or the natural environment more generally. We identified a total of 169 studies, conducted in 43 countries, across 6 continents.

What we found is that, across the board, environmental education yields several important benefits. For example, it reliably increases students’ environmental knowledge. Students learn to better understand the causes and consequences of environmental problems (e.g., climate change) and what can be done to help counter these. It also promotes environmental attitudes, such that students develop more favorable thoughts and feelings on the importance of environmental sustainability. Importantly, environmental education also promotes behavior change. For example, students who have taken part in environmental education are more likely to recycle their waste, to save energy, or to contribute to green initiatives in their schools or communities. Interestingly, from our findings, it did not seem to matter much what type of educational activity or program was offered. Having students learn about the quality of the natural environment and its protection had demonstrable benefits, regardless of whether education was provided in the context of a field trip, school-based curriculum, traditional class-based lesson, joint investigation, or a combination of these.

It is based on these and similar findings (Ardoin et al., 2018; Świątkowski et al., 2024) that my colleagues and I support calls for building teacher capacity and making environmental education part of national curricula (Sabarwal et al., 2024; United Nations Educational, Scientific and Cultural Organization, 2024; United Nations International Children’s Emergency Fund, 2021). Of course, solutions for society’s problems cannot be routinely outsourced to our schools. The schoolday is finite, and so is the expertise of educators. At the same time, schools do have a responsibility to help students navigate the world that they are part of, and prepare them for what will be the defining challenges of their time. The current situation in many countries is that environmental education is offered at the initiative of well-intentioned schoolboards or individual teachers (United Nations Educational, Scientific and Cultural Organization, 2021). This is not enough. Environmental education should be a matter of course for all youth, regardless of where they go to school. United Nations Educational, Scientific and Cultural Organization (2024) has set the ambitious goal that by 2030, environmental education should be incorporated in the national curricula of at least 90% of countries. As their Director-General, Audrey Azoulay (2024), says:

Greening schools and curricula is one of the best levers to tackle climate disruption in the long-term. It’s time to mainstream environmental education across school subjects, at all levels of education with an action-oriented approach that helps young people understand their power to make a difference.

I hope that developmental and educational scientists from around the world will help convince their countries of the importance of this message, and the scientific evidence that supports it. As societies, we will need to help youth realize their potential as engaged citizens, who are able to meaningfully contribute to sustainability transitions (cf. Crone et al., 2024; Toenders et al., 2024).

## New Directions for Promoting Pro-Environmental Behavior Change

Even if promoting pro-environmental behavior with education is possible, it is not easy, and it is especially hard with adolescents (Świątkowski et al., 2024; Thomaes et al., 2023). Traditional educational programs mainly rely on knowledge transmission, awareness building, and skill training to promote pro-environmental behavior. For example, these programs explain the consequences of rising temperatures if we continue to live our lives the way we do. Or they explain what people can do, or *should* do, to halt climate change. What students may often take from these messages is that they should refrain from engaging in inherently appealing behaviors (e.g., buying affordable clothes from a fast fashion brand, traveling by plane to a desirable holiday destination) to be able to contribute to climate mitigation. While such a message is factually correct, it can also be demotivating. Most adolescents find it hard to refrain from instantly rewarding behavior (even if they are aware of its longer-term costs), and do not like to be told what to think or do—they rather think for themselves, or create their own solutions (Steinberg, 2014; Van Petegem et al., 2015; Vansteenkiste et al., 2005).

In their analysis of the effectiveness of educational programs for promoting adolescent behavior change (e.g., programs targeting health behavior, school discipline, or aggression), Yeager and colleagues (2018) show that traditional programs (that build knowledge, awareness, and behavioral skills) often have reduced effectiveness when implemented with adolescents. This is, they argue, because many of these programs try to convince youth of a motivation for behavior change that is different from the motivation that drives their behavior in their everyday lives. The authors argue that, instead, effective educational programs and interventions align the targeted behavior with adolescents’ here-and-now priorities.

In line with this analysis, we recently developed the sustainability motive-alignment hypothesis to understand the motivational processes that underlie and find ways to promote, adolescents’ pro-environmental behavior (Thomaes et al., 2023). The hypothesis is built on the idea that adolescents will be internally motivated to engage in behavior that they think will help them pursue their personal motives: the needs, goals, and desires that they deeply care about in their daily lives. Two central adolescent motives are to pursue autonomy and peer status (Bryan et al., 2016; Galla et al., 2021; Yeager et al., 2018). Adolescents deeply care about experiencing agency over their lives, and having a say about things that matter to them. They also have a strong need to be esteemed or respected by their peers. These motives shape how adolescents make sense of and act on the world around them. They are tempted to engage in behaviors to the extent that doing so allows them to attain autonomy or garner status.

Accordingly, the motive-alignment hypothesis posits that adolescents will often fail to engage in pro-environmental behavior if they do not construe such behavior as relevant to their autonomy or status motives. In this scenario, pro-environmental behavior feels like a chore—behavior that is “right” or “expected,” but not motivating in and of itself. Conversely, the hypothesis also suggests that this scenario can be turned around. If adolescents do see how pro-environmental behavior can help them experience autonomy or status, they will be intrinsically motivated to act accordingly. In this scenario, pro-environmental behavior may feel, for example, like behavior for people who are mature enough to stand up for what they care about. Note the similarity between these hypothesized drivers of pro-environmental behavior and what young activists said about what motivates their climate activism (discussed previously): They mentioned that their activism allows them to independently express their personal values and convictions, to voice the concerns of their generation, and to stand up for social justice. Thus, I propose that similar personal motives may underlie adolescents’ climate activism and more everyday forms of pro-environmental behavior.

The motive-alignment hypothesis has implications for climate communication and education programs. It suggests that these programs may be improved by using techniques that reshape how adolescents construe pro-environmental behavior—from a low priority chore to an activity that embodies what they deeply care about. For example, in one ongoing field experiment (not yet completed at the time of writing), we test the effectiveness of motive-aligned climate communication, implemented in secondary schools, to promote students’ pro-environmental behavior. Participants are randomly assigned to one of two conditions. In the control condition, they work with traditional climate educational materials which explain the causes and consequences of climate change, and point out that we can all play our part to create a healthier planet by engaging in pro-environmental behavior (e.g., recycling, sustainable consumption). In the motive-alignment condition, participants work with similar educational materials. Yet, here, they learn about how youth their age want things to change and are increasingly taking matters into their own hands by making sustainable behavioral choices, thus protecting the planet and setting an example to other generations. The motive-alignment condition is thus designed to convey that pro-environmental behavior is autonomy- and status-relevant behavior. We will test if the motive-alignment (vs. control) condition is more effective at promoting adolescents’ sustainable food choices at school, as well as adolescents’ willingness to support environmental organizations.

## Concluding Comments

Now is a sensitive period for the emerging field of climate change and youth development, when we collectively decide both what questions we address and how we address them. One priority will be to better understand the psychological and mental health impacts of climate change on young people of different ages, both in terms of exposure to acute extreme weather events (e.g., storms), more enduring climatic impacts (e.g., droughts), and existential stressors (e.g., uncertainty about the future). In particular, a thorough understanding of the psychological processes that account for climate-related psychological vulnerability and resilience is needed to be able to develop effective intervention (Burke et al., 2018; Vergunst & Berry, 2022). Another priority will be to investigate the psychological causes and consequences of the many ways in which youth express their engagement with climate change, in addition to their private-sphere pro-environmental behavior. This may include contributing to existing political systems or institutions (e.g., contributing to climate strategies of a political party as youth representative), critiquing or acting against existing political systems or institutions (e.g., contributing to local campaigns against fossil fuel extractions), or engaging in political activism with the goal of transforming existing power structures or societal norms (e.g., supporting degrowth or climate justice movements)—forms of climate engagement and activism which have been labeled “dutiful dissent,” “disruptive dissent,” and “dangerous dissent,” respectively (O’Brien et al., 2018). Also, a priority will be to develop theory- and evidence-based climate education and communication programs that are powerful at motivating young people to actively engage with climate change, either privately or publicly. We should learn from heterogeneity in the effectiveness of such programs (i.e., when and for whom are these programs more, or less, effective?) to develop well-rounded behavior change theory and be able to provide nuanced advice to policymakers.

A final priority is to ensure that our research will be truly global. We should learn from mistakes of the past, when the majority of the world’s population was underrepresented in developmental research and the behavioral sciences more generally (Nielsen et al., 2017; Raval et al., 2023). The meteorological and psychological impacts of climate change differ across countries, and the same is true for the opportunities available to young people for engaging with climate change. What I hope is that, as a field, we will do justice to such diversity by prioritizing cross-cultural investigation and multi-site replication. Such research will be facilitated by international consortia such as Developmental Scientists for Climate Action (i.e., DevSCA; www.devsca.org)—that is, a group of researchers, educators, and practitioners from around the world that has been formed to improve our global and transdisciplinary understanding of young people’s responses to climate change and inform advocacy. Together, developmental scientists can use their expertise to support young people from across the world to cope with and help mitigate climate change.
